# Dietary Phthalate Exposure in Pregnant Women and the Impact of Consumer Practices

**DOI:** 10.3390/ijerph110606193

**Published:** 2014-06-12

**Authors:** Samantha E. Serrano, Catherine J. Karr, Noah S. Seixas, Ruby H. N. Nguyen, Emily S. Barrett, Sarah Janssen, Bruce Redmon, Shanna H. Swan, Sheela Sathyanarayana

**Affiliations:** 1 Center for Child Health, Behavior and Development, Seattle Children’s Research Institute, Seattle, WA 98121, USA; E-Mail: samserrano0202@gmail.com; 2 Department of Environmental and Occupational Health Sciences, University of Washington, Seattle, WA 98195, USA; E-Mail: ckarr@u.washington.edu; 3 Department of Pediatrics, School of Medicine, University of Washington, Seattle, WA 98195, USA; E-Mail: nseixas@u.washington.edu; 4 Department of Epidemiology, University of Minnesota, Minneapolis, MN 55455, USA; E-Mail: nguy0082@umn.edu; 5 Department of Obstetrics and Gynecology, School of Medicine and Dentistry, University of Rochester, Rochester, NY 14623, USA; E-Mail: emily_barrett@urmc.rochester.edu; 6 Department of Urology, School of Medicine, University of California San Francisco, San Francisco, CA 94143, USA; E-Mail: sarah.janssen@ucsf.edu; 7 Department of Medicine, University of Minnesota Medical School, Minneapolis, MN 55455, USA; E-Mail: redmo001@umn.edu; 8 Department of Preventive Medicine, Icahn School of Medicine at Mount Sinai, New York, NY 10029, USA; E-Mail: shanna.swan@mssm.edu

**Keywords:** food, phthalates, diet, consumer practices, behavior, ecofriendly, organic, unprocessed, prenatal exposure, pregnant women

## Abstract

Phthalates are ubiquitous endocrine-disrupting chemicals that are contaminants in food and contribute to significant dietary exposures. We examined associations between reported consumption of specific foods and beverages and first trimester urinary phthalate metabolite concentrations in 656 pregnant women within a multicenter cohort study, The Infant Development and Environment Study (TIDES), using multivariate regression analysis. We also examined whether reported use of ecofriendly and chemical-free products was associated with lower phthalate biomarker levels in comparison to not following such practices. Consumption of one additional serving of dairy per week was associated with decreases of 1% in the sum of di-2-ethylhexyl phthalate (DEHP) metabolite levels (95% CI: −2.0, −0.2). Further, participants who reported sometimes eating homegrown food had monoisobutyl phthalate (MiBP) levels that were 16.6% lower (95% CI: −29.5, −1.3) in comparison to participants in the rarely/never category. In contrast to rarely/never eating frozen fruits and vegetables, participants who reported sometimes following this practice had monobenzyl phthalate (MBzP) levels that were 21% higher (95% CI: 3.3, 41.7) than rarely/ever respondents. Future study on prenatal dietary phthalate exposure and the role of consumer product choices in reducing such exposure is needed.

## 1. Introduction

Phthalates, a family of synthetic chemicals derived from phthalic acid, are produced in high volume (over 470 million pounds per year) for use in a wide range of industrial, medical, and consumer products [1,2]. High-molecular weight phthalates, including di-2-ethylhexyl phthalate (DEHP) and benzylbutyl phthalate (BzBP), are most well-known as plasticizers, added to polyvinyl chloride (PVC) products to impart greater flexibility, durability and heat resistance. Dibutyl phthalates (DBP, DiBP) and diethyl phthalate (DEP), low-molecular weight chemicals, are primarily utilized for non-PVC applications such as adhesives, fixatives, sealants, and detergents and are found in many personal care products [1,2,3]. Due to their use as fragrance carriers, these phthalate species are frequently detected at high concentrations in perfumes and other heavily fragranced products [4].

Some phthalates exhibit anti-androgenic activity and may disrupt proper differentiation and formation of male sex organs during critical windows of development, including during the first trimester of pregnancy in humans [5,6]. Significant associations between increased maternal urinary concentrations of DEP, diisobutyl phthalate (DiBP), di-*n*-butyl phthalate (DnBP) and DEHP metabolites and shorter anogenital distance (AGD) in male infants have been demonstrated [6,7]. This is in line with animal studies that report similar associations between prenatal exposure to DnBP and DEHP, as well as BzBP and adverse male reproductive effects [5]. Prenatal exposures have also been linked to changes in timing of labor and infant and child neurobehavioral outcomes [8,9,10,11,12,13].

Because phthalates are not chemically bound to products, leaching, migration, and evaporation during use can occur, resulting in human exposure [2]. In the diet, phthalate-containing materials, particularly those made from PVC, including plastic tubing, conveyor belts, lid gaskets, gloves and packaging utilized during food processing and storage are considered important exposure sources [8,14,15]. Phthalates have also been approved for use in printing inks and adhesives on wrappers as well as paper/paperboard materials that come into contact with food [16,17]. Of note, fat content is an important factor for the contamination of food with high molecular weight phthalate species [15]. According to an exposure study in Europe, diet is a significant source of exposure to DEHP, DBP and BzBP in the general population [8]. Despite rapid elimination in urine and feces (~1–2 days), the virtually constant exposure to these chemicals results in frequent detection of metabolites in the body [18].

Effective strategies to prevent exposures in pregnant women are of great interest given the particular vulnerability of the developing fetus to reproductive impacts. Observations of an Old Order Mennonite (OOM) community from Germantown, Pennsylvania suggests that the consumption of mostly homegrown produce, the elimination of cosmetics and the limited use of personal care products (PCPs) may contribute to the significantly lower metabolite concentrations in this population compared to larger, national samples [19]. Attempts to lower phthalate exposure through diet have yielded mixed results. In one small intervention study by Rudel *et al.*, complete replacement of canned foods with fresh, mostly organic meals prepared without plastics reduced concentrations of DEHP metabolites, but a similar intervention by Sathyanarayana *et al.* was unable to replicate these results and instead reported an unexpected increase of median DEHP metabolite levels due to high contamination of spices and dairy products [20,21]. This discrepancy in results highlights the difficulty in anticipating phthalate content in food and the importance of targeting diet for exposure reducing interventions.

Little is known about how typical lifestyle and consumption habits impact exposure in pregnant women. A recent study from our group reported that pregnant women who felt that environmental chemicals were dangerous tended to follow behaviors believed to reduce exposures including choosing organic foods, foods in plastics they thought were safe and selecting for PCPs marketed as “ecofriendly”, “environmentally-friendly” or “chemical-free”. These women also reported limiting fast food intake [22]. It is unclear, however, whether variation in personal behavioral practices translate into differences in phthalate body burden.

This study aimed to (1) determine the dietary contribution of urinary phthalate metabolites among first trimester English-speaking pregnant women from four US cities and (2) determine if self-reported practices of using environmentally friendly personal care and household products and consuming chemical-free diets are associated with reduced urinary phthalate concentrations. With our second study aim we hoped to understand whether womens’ perceptions of consuming safer products would actually translate to lower exposures.

## 2. Materials and Methods

### 2.1. Study Participants

The Infant Development and Environment Study (TIDES), is a multi-center cohort study designed to examine the association between maternal phthalate exposures and infant reproductive development. The study population consists of pregnant women age 18 and over who received prenatal care and delivered at academic medical centers in Minneapolis, MN, (University of Minnesota), Rochester, NY (University of Rochester School of Medicine and Dentistry), Seattle, WA (Seattle Children’s Hospital and University of Washington School of Medicine) and San Francisco, CA (University of California at San Francisco). Women were recruited from 2010–2012. Eligibility criteria included: less than 13 weeks pregnant, English-speaking and with no serious medical conditions or threats to the pregnancy. Human subject committees at all study institutions approved TIDES and related research, and all participants signed informed consents. Subjects provided spot urine samples and completed questionnaires in each trimester of pregnancy; however the analysis for this study is based on only the first trimester survey and urine sample.

### 2.2. Questionnaires

After providing a urine sample during initial consent visits, first trimester questionnaires were completed through a secure online system, by mail, phone or in person at each participant’s convenience during the pregnancy. Information on maternal demographics, anthropometrics, food consumption frequency and behavioral practices/lifestyle that could potentially impact environmental exposures was collected.

Women reported on the number of servings of nine food groups (peanut butter (or other nut butters), beef, seafood, poultry, other meats (*i.e.*, pork, lamb), spices, oils and fats, soy, and dairy) eaten in a typical week. Participants were also asked about the number of fast food, restaurant, take-out, or home delivered meals they consumed in a typical week since becoming pregnant. Consumption of drinks in bottles and drinks in cans was reported on a daily basis within a typical week since becoming pregnant. These particular foods, meals and beverages were investigated based on the increased likelihood of phthalate contamination due to fat content, high level of processing and packaging.

Using a 5-category frequency scale (always, usually, sometimes, rarely, never) participants also indicated how often they purchased ecofriendly, chemical-free and environmentally friendly personal care and household products and organic food as well as the frequency of consuming foods and beverages they believed to be in safe plastics and of checking recycling codes on bottles. Ecofriendly was defined as “not harmful to the earth and its inhabitants” in an attachment to the questionnaire. Participants were not provided with definitions for the terms “chemical-free” or “environmentally friendly.” These questions were phrased in a vague manner to capture a range of practices that women personally believed to reduce exposure to harmful chemicals.

Using a 6-category frequency scale (always, usually, sometimes, rarely, never, don’t know), women also reported on how often the food they consumed during pregnancy was (1) grown, raised or caught by individual/family/friends; (2) unprocessed; (3) canned fruits and vegetables; (4) frozen fruits and vegetables; (5) fresh fruits and vegetables; or (6) marked as “organic”, “pesticide-free”, “chemical-free”. Participants were asked to report on the frequency of following an organic diet in a typical setting as well as since becoming pregnant. A copy of these questions and responses is provided as a supplemental material.

### 2.3. Biospecimens

First trimester urine was collected in sterile and phthalate-free specimen cups during initial recruitment visits, transferred to cryovials, and stored in freezers at <−80 °C. We measured specific gravity using a handheld refractometer at the time of urine collection, which was calibrated with deionized water before each measurement. Phthalate metabolite concentrations were analyzed at two different sites. Three hundred subject samples were analyzed at the Environmental Health Laboratory at the University of Washington (UW). Per a modified version of the CDC method 6306.03, glucoronidated phthalate monoesters underwent enzymatic deconjugation, followed by online-solid phase extraction (SPE) coupled with reversed high performance liquid chromatography-electrospray ionization-tandem mass spectrometry (HPLC-ESI-MS/MS) to quantify the simple monoesters in urine [23]. An additional 369 subject samples were analyzed by the Division of Laboratory Sciences, National Center for Environmental Health, Center for Disease Control and Prevention (CDC). At CDC, urine samples were analyzed using a modified method described in Silva *et al.* 2007 that involved the enzymatic deconjugation of the phthalate metabolites from their glucuronidated form, automated on-line sold phase extraction, separation with high performance liquid chromatography and detection by isotope-dilution tandem mass spectrometry [24]. The eight phthalate metabolites that were measured in urine and their five corresponding parent compounds are shown in Table 1. We selected these particular phthalate species based on their biological relevance demonstrated in animal and human data.

Process and instrument blanks as well as field blanks were run in each lab for quality assurance of analytical and sampling procedures. For the field blank collection, deionized water was purchased, poured into phthalate-free urine cups and transferred with disposable pipettes to 5 mL cryovials. These blanks were then interspersed with subject samples to be shipped to laboratories. Ten urine samples were also analyzed at both UW and the CDC for comparison. Geometric mean urinary phthalate metabolite concentrations were compared to the US female population of reproductive age from the National Health and Nutrition Examination Survey (NHANES) 2011–2012 [25].

**Table 1:** Phthalate parent compounds and their metabolites.

Phthalate Name	Abbreviation	Urinary Metabolite	Abbreviation
Diethyl phthalate	DEP	Monoethyl phthalate	MEP
Di-isobutyl phthalate	DiBP	Monoisobutyl phthalate	MiBP
Di-*n*-butyl phthalate	DnBP	Mono-*n*-butyl phthalate	MnBP
Benzylbutyl phthalate	BzBP	Monobenzyl phthalate	MBzP
Di-2-ethylhexyl phthalate	DEHP	Mono-2-ethylhexyl phthalate	MEHP
		Mono-(2-ethyl-5-hydroxyhexyl) phthalate	MEHHP
		Mono-(2-ethyl-5-oxohexyl) phthalate	MEOHP
		Mono-(2-ethyl-5-carboxypentyl) phthalate	MECPP

### 2.4. Statistical Analysis

The limit of detection (LOD) of metabolites was between 0.2 and 2.0 ng/mL for the UW samples and 0.2 and 0.6 ng/mL for the CDC samples. For concentrations below the LOD, a value equal to each sample’s specific LOD divided by the square root of 2 was used [26]. All urinary phthalate metabolite levels were adjusted for dilution using specific gravity measurements and logarithmically transformed (natural log) to normalize distributions [27]. To calculate the molar sum of the DEHP metabolites, MEHP, MEHHP, MEOHP and MECPP were divided by their molecular weights and added (∑ DEHP metabolites = (MEHP/278) + (MEHHP/294) + (MEOHP/292) + (MECPP/308)) × 1,000).

Each food consumption variable was roughly divided into three equal categories and analyzed both continuously and categorically. We performed categorical analysis to compare effect size with continuous analysis and potential non-linear effects. Further, responses of always, usually, sometimes, rarely, and never were reduced to three categories (always/usually, sometimes, and rarely/never) while responses of don’t know were considered missing information. We performed bivariate linear regression analysis for the various food groups and consumption patterns and log-transformed urinary phthalate metabolite concentrations. For each association that was statistically significant (*p* < 0.05) for at least one of the phthalate metabolite species, we constructed a multiple linear regression model.

It was determined *a priori* that maternal age, BMI, race and education would be included in multiple regression analysis given the significant associations with phthalate metabolite levels, diet and/or consumer behaviors reported in previous studies [22,28,29,30]. We conducted bivariate linear regression analyses for other demographic characteristics (study center, income) and log-transformed urinary phthalate metabolite levels; demographic characteristics that were found to have statistically significant (*p* *<* 0.05) associations with at least one of the phthalate metabolite species and were not highly correlated (spearman rank correlation coefficient <0.6) with other covariates were included in all multiple regression models.

The main predictor in each multiple regression model was an individual food group or consumption practice variable and covariates included maternal age, BMI, race, study center, and education. Outcomes were urine specific gravity (USG)-adjusted, log-transformed phthalate metabolite concentrations. Associations with a *p*-value less than 0.05 were considered statistically significant. In categorical analysis, we performed a test of trend by including the categorical characteristic as a ordinal linear variable in the model. We subsequently examined residual diagnostics and potential outliers for each model and compared results with and without outliers as a sensitivity analysis. All data analyses were performed with STATA software version 12 (College Station, TX, USA). In order to more easily interpret multiple linear regression results in the text, we converted beta coefficients to percentages with the following equation: % = (e^β^ − 1) × 100.

## 3. Results

### 3.1. Population Characteristics

In total, 969 women enrolled and 894 women (92%) provided first-trimester questionnaire data. Seventy consented women dropped out before completing the first trimester questionnaire. At the time of this particular analysis, urine samples were available for 669 participants. All subjects that provided a questionnaire, urine sample and had complete covariate data were included in the final dataset (*n* = 656). Overall, participants were primarily white (70.1%) and had some college/graduate education or a college/graduate degree (86.7%). The mean age was 31 years (min–max: 18–44) and mean BMI was 26.3 (min–max: 14.1–61.1) (Table 2).

**Table 2:** Characteristics of pregnant women within TIDES cohort (*n* = 656).

Characteristic	No.	%
**Age (years)**		
<20	22	3.4
21–30	281	42.8
31–40	337	51.4
>40	16	2.4
**BMI (kg/m^2^)**		
Underweight	9	1.4
Normal	344	52.4
Overweight	154	23.5
Obese	149	22.7
**Study Center**		
San Francisco, CA	155	23.6
Minneapolis, MN	180	27.4
Rochester, NY	187	28.5
Seattle, WA	134	20.4
**Race**		
White	460	70.1
Asian	44	6.7
Black/African American	83	12.7
Other	45	6.9
More than one race	24	3.7
**Education**		
High school or less	87	13.3
Some college/tech. school or college/tech. school graduate	283	43.1
Some graduate school or graduate degree	286	43.6
**Urine Specific Gravity**		
Mean (range)	1.01 (1.00–1.12)	

### 3.2. Food Consumption and Consumer Practices

Of the food groups assessed, only peanut butter, spices, oils, butter, lard, shortening, dairy, fast food, and drinks in plastic bottles were significantly associated with phthalate metabolite levels. Dairy was consumed at the highest rate at an average of 10.7 servings per week (range: 0–40). Average consumption of fast food was reported at one meal per week (range: 0–30) (Table 3).

Almost half of participants (45.4%) reported usually or always purchasing personal care products (PCPs) that were ecofriendly, chemical-free or environmentally friendly. Similar observations were made for participants that reported usually or always consuming food that was organic, ecofriendly, and chemical-free (47.4%). More than half of respondents reported rarely or never consuming canned (79.7%) or frozen fruits and vegetables (56.6%). In contrast, usually or always eating food grown, raised, or caught by the woman (or her family/friends) was only reported by 2.9% of the population (Table 4).

**Table 3:** Reported dietary intake of foods by pregnant women within TIDES cohort (*n* = 656).

Food Item	Frequency (Servings/Day or Week)	No.	Percent
Peanut butter (or other nut butters)	Mean (range)	2.3 (0–30)	
	0/week	175	26.7
	1–2/week	255	38.9
	≥3/week	226	34.5
Spices	Mean (range)	8.5 (0–30)	
	0–5/week	212	32.3
	6–9/week	197	30.0
	≥10/week	247	37.7
Oils, butter, lard, shortening	Mean (range)	6.5 (0–30)	
	0–3/week	188	28.7
	4–6/week	172	26.2
	≥7/week	296	45.1
Dairy products	Mean (range)	10.7 (0–40)	
	0–6/week	171	26.1
	7–13/week	249	38.0
	≥14/week	236	36.0
Fast food	Mean (range)	1.0 (0–30)	
	0/week	357	54.4
	1/week	153	23.3
	≥2/week	146	22.3
Beverage in plastic bottle	Mean (range)	2.7 (0–32)	
	0/day	133	20.3
	1–2/day	283	43.1
	≥3/day	240	36.6

**Table 4:** Distribution of the number of participants following nine consumer practices (count, %).

Consumer Practice	*n*	Always/Usually	Sometimes	Rarely/Never
**Typically, participant tries to make sure that**				
Personal care products are ecofriendly, chemical-free or environmentally friendly	656	298 (45.4)	224 (34.2)	134 (20.4)
Household products are ecofriendly, chemical-free or environmentally friendly	655	267 (40.7)	233 (35.5)	155 (23.6)
Food is organic, ecofriendly, chemical-free or environmentally-friendly	656	311 (47.4)	205 (31.3)	140 (21.3)
Checks recycling code on bottles	656	120 (18.3)	124 (18.9)	412 (62.8)
**Since becoming pregnant**				
Food consumed is grown, raised, caught by individual or family/friends	646	19 (2.9)	110 (17.0)	517 (80.0)
Food consumed is unprocessed	637	279 (43.8)	251 (39.4)	107 (16.8)
Fruits/vegetables consumed are canned	655	28 (4.3)	105 (16.0)	522 (79.7)
Fruits/vegetables consumed are frozen	654	35 (5.4)	249 (38.1)	370 (56.6)
Food consumed is marked “organic” “pesticide-free” or “chemical-free”	612	253 (41.3)	214 (35.0)	145 (23.7)

### 3.3. Analysis of Urinary Phthalate Metabolite Concentrations

We found two of 13 field blanks from the UW site with levels of MnBP above the limit of detection (13 and 15 ng/mL). These particular field blanks were considered non-representative of study sample processing based on discrepancies with the collection protocol and were therefore discarded. Further, the 10 samples analyzed at both laboratories differed only between 0.6% and 31% and were found to be comparable based on statistically significant Pearson correlation coefficients greater than 0.7.

Of the phthalate metabolites assayed, over 65% of women had MEHP and MBzP concentrations above the LOD and over 92% had MEP, MiBP, MnBP, MEHHP, MEOHP and MECPP concentrations above the LOD (Table 5). Of the single monoesters, MEP had the highest geometric mean concentration in the population (unadjusted: 27.9 ng/mL (range: 24.6–31.5); adjusted: 35.6 ng/mL (range: 32.1–39.6)). Table 5 demonstrates that the study population was comparable for all metabolite levels to the U.S. female population of reproductive age from the most recently available National Health and Nutrition Examination Survey (NHANES) 2011–2012 [25].

### 3.4. Maternal Factors and Urinary Phthalate Metabolite Concentrations

The bivariate relationships between phthalate biomarkers and maternal characteristics were in line with results from other studies (Table 6). Increasing maternal age was associated with lower concentrations of all phthalate metabolites. Additionally, maternal BMI was positively associated with MEP, MiBP, MnBP, MBzP but not the DEHP summary measurement. Participants in Rochester tended to have greater concentrations of all phthalate metabolites as compared to participants in San Francisco. Finally, having a high school education or less was associated with higher concentrations of all metabolites except for DEHP summary measures in comparison to having a graduate school education.

### 3.5. Food Consumption and Urinary Phthalate Metabolite Concentrations

Using continuous data in multiple regression analysis, we found the boldest associations between one additional serving of spices per week and lower MiBP, MnBP and MBzP concentrations ranging between approximately 1% (−2.3, 0.2) and 2% (95% CI: −3.2, −0.4). Similarly, MiBP concentrations were 2% lower with one additional serving of dairy per week (95% CI: −2.0, −0.4) while sum of DEHP metabolite levels were lower by 1% (95% CI: −2.0, −0.20). Running models without outliers did not impact these results.

With analysis of categorical data, MiBP, MnBP, MBzP and the sum of DEHP metabolite concentrations were between 17.5% (95% CI: −29.8, −3.1) and 21.9% (95% CI: −35.5, −5.4) lower in participants reporting consumption of 10 or more servings of spices per week (highest exposure category) in comparison to 0–5 servings per week (lowest exposure) (Table 7). We observed trends of decreasing concentrations of MiBP (*p* = 0.01), MnBP (*p* = 0.01), MBzP (*p* = 0.01) and the sum of DEHP metabolites (*p* = 0.02) with increasing servings of spices. Additionally, consumption of 14 or more servings of dairy per week was associated with lower MiBP levels by 23.3% (95% CI: −35.5, −8.7) in comparison to 0–7 servings per week. The test for trend showed decreases of MiBP levels across increasing categories of dairy consumption (*p* = 0.002). We observed the boldest association between the medium exposure category of seven to 13 servings per week of dairy and higher MEP levels of 35.9% (95% CI: 4.1, 77.5) in comparison to 0–6 servings per week. No statistically significant associations were found between consumption of peanut butter, oils, butter, lard, fast food and drinks in bottles and urinary phthalate metabolites (Table 7).

### 3.6. Consumer Practices and Urinary Phthalate Metabolite Concentrations

In multiple regression analysis, we observed the greatest differences in phthalate concentrations for MEP in relation to various consumer practices. Women who reported rarely/never purchasing ecofriendly, chemical-free and environmentally friendly personal care (PCP) and household products had higher levels of MEP by 53.6% (PCPs 95% CI: 16.6, 102.2) and 32.9% (household products 95% CI: 1.6, 73.9) compared to always/often respondents. Sometimes following these practices were associated with MiBP levels that were 17.6% (PCPs 95% CI: −28.4, −5.1) and 18% (household products 95% CI: −29.0, −5.3) lower as compared to always/usually respondents. We observed trends for increasing concentrations of MEP with decreasing frequency of purchasing ecofriendly PCPs (*p* = 0.01) or house products (*p* = 0.03). There were no significant associations between PCPs and household products and MnBP, MBzP or summed DEHP metabolites. In regards to diet, reports of rarely/never eating food that is organic, ecofriendly, environmentally-friendly or chemical-free were related to lower MEP levels by 46.5% (95% CI: 9.3%, 96.5%) as compared to always/usually following this practice. We also found that participants who reported sometimes eating food that is grown, raised, or caught during pregnancy had MEP levels that were 26.3% (95% CI: −44.0, −2.8) lower and MiBP levels that were 16.6% (95% CI: −29.5, −1.3) lower than participants in the rarely/never category. In comparison to rarely/never eating frozen fruits and vegetables during pregnancy, participants who reported sometimes following this practice had higher MBzP levels by 21% (95% CI: 3.3, 41.7). Lastly, rarely or never eating food marked “organic”, “pesticide-free”, or “chemical-free” during pregnancy was associated with sum of DEHP metabolite levels that were 17.7% (95% CI: −31.8, −0.7) lower in comparison to those in the always/usually category. No statistically significant associations were found between consuming unprocessed foods, canned fruits and vegetables and checking recycling codes on bottles and urinary phthalate metabolite levels (Table 8).

## 4. Discussion

We examined the association between consumption of significant food groups and phthalate metabolite concentrations in a cohort of women in the first trimester of pregnancy, a critical window for the developing reproductive tract of the fetus. With analysis of continuous data, we did not find that any particular food group significantly contributed to exposure but instead observed negative associations between consumption of spices and dairy with some phthalate metabolite levels. Of great surprise was the negative association between dairy intake and levels of DEHP metabolites despite the well-known fact that foods high in fat are contaminated by higher weight phthalates that are more lipophilic, particularly DEHP.

**Table 5:** Geometric mean phthalate metabolite concentrations in pregnant women within TIDES cohort (ng/mL) (*n* = 656).

Parent	Metabolite	LOD (CDC)	LOD (UW) ^a^	% >LOD	USG-Adjusted Geometric Mean (95% CI) ^b^	Unadjusted Geometric Mean (95% CI)	NHANES 2011–2012 ^c^
DEP	MEP	0.6	1	99.1	35.63 (32.09–39.56)	27.85 (24.61–31.50)	33.36 (23.85–46.64)
DiBP	MiBP	0.2	0.2	97.0	5.08 (4.76–5.42)	3.97 (3.58–4.40)	5.76 (4.60–7.23)
DnBP	MnBP	0.4	1 or 2	92.1	8.23 (7.69–8.82)	6.43 (5.81–7.13)	6.83 (5.06–9.22)
BzBP	MBzP	0.3	1	86.9	4.28 (3.94–4.64)	3.34 (3.00–3.73)	4.20 (3.39–5.20)
DEHP	MEHP	0.5	1	65.7	2.54 (2.37–2.73)	1.99 (1.81–2.18)	1.61 (1.38–1.89)
	MEHHP	0.2	0.2 or 1	96.8	7.68 (7.16–8.25)	6.01 (5.43–6.64)	7.43 (6.34–8.71)
	MEOHP	0.2	1	97.1	5.56 (5.19–5.95)	4.34 (3.94–4.79)	5.12 (4.38–5.98)
	MECPP	0.2	0.2 or 1	97.1	10.05 (9.41–10.72)	7.85 (7.14–8.64)	12.09 (10.45–13.98)
	∑ DEHP Metabolites ^d^				90.96 (85.35–96.95)	71.09 (64.73–78.08)	N/A

Notes: **^a^** Samples were analyzed in 2 batches. Two LODs reported due to differences in instrument sensitivity and/or level of background; **^b^** Adjusted for urine specific gravity; **^c^** Female population of reproductive age (20–40 years) values calculated from Centers for Disease Control and Prevention (CDC). National Center for Health Statistics (NCHS). National Health and Nutrition Examination Survey Data. US Department of Health and Human Services, Centers for Disease Control and Prevention, 2011–2012 [25]; unadjusted weighted geometric means.; **^d^** ∑ DEHP Metabolites is the sum of each metabolite divided by its molecular weight (MEHP, MEHHP, MEOHP, MECPP); * 1,000 reported in nnmol/L;

**Table 6:** Bivariate relationships between participant characteristics and phthalate metabolite concentrations (*n* = 656) (percent change estimate, 95% CI).

Characteristic	MEP	MiBP	MnBP	MBzP	∑DEHP Metabolites
Maternal Age	−4.9 * (−6.6, −3.1)	−3.2 * (−4.3, −2.1)	−3.7 * (−4.9, −2.5)	−6.2 * (−7.5, −4.9)	−1.9 * (−3.0, −0.8)
BMI	1.9 * (0.2, 3.6)	1.7 * (0.7, 2.8)	2.2 * (1.1, 3.3)	2.7 * (1.4, 4.0)	0.8 (−0.2, 1.8)
*Race/Ethnicity*					
White	ref	ref	ref	ref	ref
Asian	−6.1 (−37.9, 42.0)	16.9 (−9.4, 50.8)	18.8 (−9.1, 55.3)	−20.9 (−42.4, 8.7)	7.1 (−17.1, 38.4)
Black/African-American	116.9 * (58.7, 196.4)	111.0 * (74.1, 155.8)	95.0 * (59.3, 138.8)	98.7 * (56.4, 152.5)	31.7 * (8.5, 59.9)
Other	94.4 * (29.2, 192.6)	26.1 (−2.0, 62.3)	43.0 * (9.7, 86.5)	45.6 * (6.4, 99.3)	−0.5 (−22.8, 28.2)
More than one race	23.8 (−28.4, 114.3)	24.2 (−11.4, 74.1)	−1.0 (−30.6, 41.2)	−11.5 (−41.9, 34.8)	−0.07 (−28.9, 40.4)
*Study Center*					
San Francisco	ref	ref	ref	ref	ref
Minneapolis	36.3 * (2.2, 81.8)	20.1 * (0.5, 43.5)	16.4 (−3.3, 40.2)	48.7 * (19.7, 84.8)	27.9 * (7.2, 52.6)
Rochester	103.2 * (52.7, 170.3)	71.3 * (43.6, 104.3)	79.0 * (48.9, 115.1)	127.7 * (83.6, 182.4)	38.4 * (16.1, 64.9)
Seattle	20.3 (−11.8, 64.0)	−2.6 (−19.6, 17.9)	6.5 (−12.8, 30.0)	33.4 * (5.6, 68.5)	35.3 * (11.9, 63.7)
*Education*					
High school or less	142.4 * (76.0, 233.8)	96.4 * (60.9, 139.6)	110.0 * (70.9, 158.6)	190.0 * (128.2, 268.4)	15.4 (−5.5, 40.8)
Some college/tech. school or graduate	48.3 * (19.1, 84.7)	6.4 (−7.1, 22.0)	15.4 * (0.2, 33.0)	18.4 * (0.5, 39.5)	2.7 (−10.4, 17.8)
Some graduate work or graduate degree	ref	ref	ref	ref	ref

Notes: * Indicates *p* < 0.05; ref = reference group for comparison in statistical analysis.

**Table 7:** Multiple regression analysis results for dietary intake and phthalate metabolite concentrations for continuous and categorical data (percent change estimate, 95% CI) **^a^**.

Food Type	Number of Servings	*n*	MEP	MiBP	MnBP	MBzP	∑DEHP Metabolites
**Peanut Butter**	servings/week	656	0.6 (−3.0, 4.4)	−1.5 (−3.7, 0.7)	2.1 (−0.3, 4.5)	1.2 (−1.5, 4.0)	0.7 (−1.6, 3.0)
	0 servings/week	175	ref	ref	ref	ref	ref
	1–2 servings/week	255	−13.2 (−33.2, 12.7)	−6.7 (−20.5, 9.4)	5.8 (−10.5, 25.1)	0.5 (−17.0, 21.8)	−7.1 (−21.0, 9.2)
	≥3servings/week	226	−14.1 (−34.8, 13.1)	−15.3 (−28.4, 0.2)	−1.9 (−17.7, 17.0)	1.6 (−17.0, 24.4)	−0.2 (−15.8, 18.4)
	*p*_trend_		*p* = 0.30	*p* = 0.05	*p* = 0.76	*p* = 0.87	*p* = 0.93
**Spices**	servings/week	656	−1.4 (−3.4, 0.6)	−1.6 * (−2.8, −0.4)	−1.7 * (−3.0, −0.4)	−1.8 * (−3.2, −0.3)	−1.0 (−2.3, 0.2)
	0–5 servings/week	212	ref	ref	ref	ref	ref
	6–9 servings/week	197	19.2 (−8.3, 55.0)	−15.1 * (−27.6, −0.3)	−0.2 (−15.6, 18.0	−11.2 (−26.7, 7.6)	−14.9 (−27.7, 0.04)
	≥10 servings/week	247	−6.5 (−28.0, 21.4)	−20.0 * (−31.8, −6.2)	−19.7 * (−32.0, −5.2)	−21.9 * (−35.5, −5.4)	−17.5 * (−29.8, −3.1)
	*p*_trend_		*p* = 0.57	*p* = 0.01	*p* = 0.01	*p* = 0.01	*p* = 0.02
**Oils, Butter, Lard, Shortening**	servings/week	656	−1.3 (−3.6, 1.1)	−0.1 (−1.5, 1.4)	−0.9 (−2.4, 0.6)	−0.6 (−2.3, 1.1)	0.1 (−1.3, 1.6)
	0–3 servings/week	188	ref	ref	ref	ref	ref
	4–6 servings/week	172	21.1 (−8.3, 59.9)	5.0 (−11.5, 24.6)	−8.6 (−23.5, 9.3)	−1.1 (−19.4, 21.4)	−1.8 (−17.4, 16.8)
	≥7 servings/week	296	−14.1 (−34.0, 11.7)	−2.1 (−16.7, 15.1)	−7.1 (−21.6, 10.0)	−4.2 (−21.1, 16.4)	3.2 (−12.4, 21.7)
	*p*_trend_		*p* = 0.17	*p* = 0.74	*p* = 0.43	*p* = 0.66	*p* = 0.67
**Dairy Products**	servings/week	656	−0.4 (−2.2, 1.3)	−1.5 * (−2.5, −0.4)	−0.9 (−2.0, 0.2)	−0.04 (−1.3, 1.2)	−1.3 * (−2.3, −0.2)
	0–6 servings/week	171	ref	ref	ref	ref	ref
	7–13 servings/week	249	35.9 * (4.1, 77.5)	−5.5 (−19.7, 11.2)	2.2 (−13.9, 21.3)	7.2 (−11.9, 30.5)	−4.8 (−19.4, 12.3)
	≥14 servings/week	236	2.8 (−22.7, 36.7)	−23.3 * (−35.5, −8.7)	−10.5 (−25.4, 7.5)	6.4 (−13.8, 31.2)	−15.5 (−29.2, 0.9)
	*p*_trend_		*p* = 0.92	*p* = 0.002	*p* = 0.19	*p* = 0.60	*p* = 0.05
**Fast Food**	servings/week	656	0.5 (−5.3, 6.8)	1.2 (−2.4, 5.0)	−0.2 (−4.0, 3.7)	−0.4 (−4.7, 4.1)	1.1 (−2.6, 4.9)
	0 servings/week	357	ref	ref	ref	ref	ref
	1 serving/week	153	10.1 (−15.4, 43.3)	−5.6 (−19.6, 11.0)	0.2 (−15.3, 18.6)	10.2 (−9.1, 33.6)	1.4 (−13.9, 19.4)
	≥2 servings/week	146	−6.6 (−30.9, 26.2)	−9.6 (−24.8, 8.7)	−16.3 (−30.9, 1.5)	−14.4 (−31.3, 6.7)	−0.4 (−17.4, 20.1)
	*p*_trend_		*p* = 0.82	*p* = 0.26	*p* = 0.11	*p* = 0.32	*p* = 1.0
**Drinks in Plastic Bottles**	drinks/day	656	0.4 (−2.9, 3.8)	−1.0 (−3.0, 1.1)	−0.8 (−2.9, 1.3)	0.3 (−2.1, 2.8)	−0.02 (−2.1, 2.1)
	0 drinks/day	133	ref	ref	ref	ref	ref
	1–2 drinks/day	283	2.9 (−22.0, 35.7)	−7.2 (−21.6, 10.0)	4.2 (−12.8, 24.4)	−7.6 (−24.6, 13.1)	2.6 (−13.6, 21.8)
	≥3 drinks/day	240	12.3 (−17.2, 52.1)	−0.02 (−0.21, 0.16)	3.9 (−14.4, 26.3)	9.8 (−12.1, 37.1)	12.7 (−6.6, 36.1)
	*p*_trend_		*p* = 0.43	*p* = 0.91	*p* = 0.73	*p* = 0.30	*p* = 0.18

Notes: ^**a**^ All models adjusted for maternal age, BMI, study center, race, and education; * Indicates *p* < 0.05; ref = reference group for comparison in statistical analysis.

**Table 8:** Multiple regression analysis results for consumption practices and phthalate metabolite concentrations (percent change estimate, 95% CI) **^a^**.

Consumption Practice	*n*	MEP	MiBP	MnBP	MBzP	∑DEHP Metabolites
**Personal care products purchased are ecofriendly, chemical-free, environmentally friendly**						
Always/Usually	298	ref	ref	ref	ref	ref
Sometimes	224	2.3 (−18.7, 14.8)	−17.6 * (−28.4, −5.1)	−12.1 (−24.2, 1.8)	−10.9 (−24.8, 5.5)	−0.6 (−13.9, 14.8)
Rarely/Never	134	53.6 * (16.6, 102.2)	−4.2 (−19.1, 13.4)	4.8 (−12.2, 25.0)	10.6 (−9.7, 35.5)	−7.2 (−21.9, 10.2)
*p*_trend_		*p* = 0.01	*p* = 0.27	*p* = 0.96	*p* = 0.60	*p* = 0.45
**Household products purchased are ecofriendly, chemical-free, environmentally friendly**						
Always/Usually	267	ref	ref	ref	ref	ref
Sometimes	233	−7.2 (−26.7, 17.3)	−18.0 * (−29.0, −5.3)	−10.5 (−23.0, 4.1)	−10.6 (−24.8, 6.2)	−9.7 (−22.0, 4.6)
Rarely/Never	155	32.9 * (1.6, 73.9)	−6.0 (−20.3, 10.8)	−1.4 (−17.1, 17.2)	13.3 (−7.0, 38.1)	−6.9 (−21.3, 10.0)
*p*_trend_		*p* = 0.07	*p* = 0.26	*p* = 0.71	*p* = 0.36	*p* = 0.32
**Food consumed is organic, ecofriendly, chemical-free, environmentally friendly**						
Always/Usually	311	ref	ref	ref	ref	ref
Sometimes	205	−5.3 (−25.8, 20.6)	−8.0 (−20.8, 6.8)	−9.4 (−22.4, 5.9)	−1.23 (−17.4, 18.1)	1.2 (−13.0, 17.8)
Rarely/Never	140	46.5 * (9.3, 96.5)	−4.1 (−20.0, 14.9)	11.8 (−7.4, 35.0)	17.5 (−5.4, 45.9)	−9.2 (−24.4, 9.0)
*p*_trend_		*p* = 0.03	*p* = 0.53	*p* = 0.43	*p* = 0.20	*p* = 0.38
**Individual checks recycling code on bottles**						
Always/Usually	120	−5.2 (−27.8, 24.5)	−9.0 (−22.9, 7.6)	−3.1 (−18.7, 15.3)	−8.3 (−24.9, 12.0)	1.8 (−14.0, 20.6)
Sometimes	124	8.8 (−16.8, 42.2)	0.7 (−14.6, 18.6)	2.0 (−14.1, 21.1)	−3.7 (−20.9, 17.2)	12.5 (−4.7, 32.9)
Rarely/Never	412	ref	ref	ref	ref	ref
*p*_trend_		*p* = 0.85	*p* = 0.33	*p* = 0.78	*p* = 0.38	*p* = 0.57
**Food consumed during pregnancy is grown, raise, or caught**						
Always/Usually	19	8.8 (−41.4, 101.8)	45.2 (−0.4, 111.8)	29.9 (−12.5, 92.6)	12.5 (−28.4, 77.0)	4.3 (−28.9, 53.0)
Sometimes	110	−26.3 * (−44.0, −2.8)	−16.6 * (−29.5, −1.3)	−11.0 (−25.4, 6.1)	−4.1 (−21.7, 17.3)	17.6 (−0.9, 39.5)
Rarely/Never	517	ref	ref	ref	ref	ref
*p*_trend_		*p* = 0.18	*p* = 0.78	*p* = 0.94	*p* = 0.96	*p* = 0.14
**Food consumed during pregnancy is unprocessed**						
Always/Usually	279	ref	ref	ref	ref	ref
Sometimes	251	22.6 (−2.9, 54.8)	8.1 (−6.3, 24.7)	9.8 (−5.5, 27.5)	−9.8 (−23.8, 6.8)	−5.2 (−17.8, 9.3)
Rarely/Never	107	0.8 (−26.8, 38.9)	7.0 (−12.1, 30.2)	12.9 (−8.1, 38.6)	5.6 (−16.2, 33.2)	2.8 (−15.5, 25.0)
*p*_trend_		*p* = 0.59	*p* = 0.38	*p* = 0.19	*p* = 0.97	*p* = 0.97
**Fruits/vegetables consumed during pregnancy are canned**						
Always/Usually	28	−29.5 (−59.3, 22.3)	15.9 (−17.2, 62.4)	2.3 (−28.1, 45.6)	31.9 (−11.9, 97.5)	−2.5 (−30.7, 37.3)
Sometimes	105	−9.1 (−32.1, 21.6)	12.6 (−5.8, 34.5)	−2.7 (−19.3, 17.3)	−3.3 (−22.0, 19.7)	−3.5 (−19.5, 15.7)
Rarely/Never	522	ref	ref	ref	ref	ref
*p*_trend_		*p* = 0.20	*p* = 0.17	*p* = 0.89	*p* = 0.48	*p* = 0.73
**Fruits/vegetables consumed during pregnancy are frozen**						
Always/Usually	35	5.3 (−34.1, 68.1)	3.9 (−21.9, 38.4)	−14.8 (−36.8, 14.9)	−8.0 (−34.6, 29.4)	−10.5 (−33.0, 19.5)
Sometimes	249	−6.2 (−24.5, 16.4)	−5.7 (−17.4, 7.7)	1.6 (−11.6, 16.7)	21.0 * (3.3, 41.7)	−11.0 (−22.2, 1.7)
Rarely/Never	370	ref	ref	ref	ref	ref
*p*_trend_		*p* = 0.72	*p* = 0.64	*p* = 0.57	*p* = 0.20	*p* = 0.10
**Food consumed during pregnancy is marked “organic” “pesticide-free” “chemical-free”**						
Always/Usually	253	ref	ref	ref	ref	ref
Sometimes	214	−15.3 (−33.9, 8.7)	12.7 (−3.5, 31.7)	4.9 (−10.7, 23.3)	7.9 (−10.1, 29.6)	3.0 (−11.8, 20.4)
Rarely/Never	145	−3.7 (−28.7, 30.1)	−5.4 (−21.6, 14.1)	−2.8 (−20.0, 18.1)	4.2 (−16.4, 30.0)	−17.7 * (−31.8, −0.7)
*p*_trend_		*p* = 0.67	*p* = 0.83	*p* = 0.91	*p* = 0.62	*p* = 0.08

Notes: ^**a**^ All models adjusted for maternal age, BMI, study center, race and education; * Indicates *p* < 0.05; ref = reference group for comparison in statistical analysis.

A review of food monitoring data, however, revealed that DEHP contamination levels in dairy vary greatly based on product type. For example, across studies, DEHP was generally found in lower concentrations in yogurt (0–102 µg/kg), skim milk (20–25 µg/kg) and low fat milk (20–50 µg/kg) while cream (180–2,700 µg/kg), cheese (41–16,800 µg/kg) and whole milk (35–2,260 µg/kg) had up to 100 times higher concentrations [8,31,32]. It is possible that dairy was associated with decreased levels of DEHP metabolites in this study population because it was not possible to differentiate between women consuming dairy products containing low versus high DEHP concentrations. In categorical analysis, we observed a strong association between the medium exposure category of consumption of dairy and higher MEP levels. However, the food monitoring data does not support this observation as concentrations tend to be low (or not detectable) across various types of dairy products [8].

In contrast to these results, a cross-sectional study by Colacino *et al.* that examined the contribution of different food types to phthalate exposure in a nationally representative sample of 2,384 individuals (ages 6–85) found poultry and egg consumption to be significantly associated with higher DEHP metabolite levels [33]. A similar study conducted by Trasande *et al.* reported that higher consumption of discretionary solid fat, meats and caloric intake was associated with increased high-molecular weight metabolite and sum DEHP metabolite levels in children and adolescents [34]. Inverse associations were observed between sum DEHP metabolites and consumption of fruit in both of these studies. Further, both studies reported increased MEP levels with intake of vegetables [33,34].

Because phthalates have very short half-lives with nearly complete elimination in urine within 24 to 48 h, Colacino *et al.* and Trasande *et al.* utilized 24 h recalls to assess dietary intakes against biomarkers 3,34]. In our study, food frequency surveys, which are typically used to determine long-term patterns of consumption, may not have provided accurate measures of dietary exposure as estimated by a spot urine sample resulting in null observations. The null results we observed may also be a reflection of the 20%–50% decline in DnBP, BBzP, and DEHP urinary metabolite concentrations in the United States in the last decade [35]. Further, DiNP and DiDP have seen an increase in use and may now be a major contaminant in food rather than the other phthalates as expected [33].

We also examined women’s self-reported typical consumption practices with urinary phthalate exposures. We found that women who reported rarely or never using ecofriendly and chemical-free personal care and household products had significantly higher MEP levels compared to women who usually or always used such products. Given that of the phthalate esters, bioburden of MEP is typically highest, (and is higher in females than males), careful choice of personal care and household products may represent a potential avenue for exposure reduction [28].

Following dietary consumption practices that promote organic, chemical-free, ecofriendly and homegrown food products was also related to lower MEP levels. DEP, the parent compound of MEP, has not typically been considered a significant food contaminant in comparison to other phthalates. However, as noted previously, Colacino *et al.* and Trasande *et al.* showed statistically significant positive associations between intake of vegetables and the metabolite of DEP, suggesting that this particular food group may also be an important exposure source for DEP [33,34].

Participants who reported sometimes eating food that is grown, raised, or caught since becoming pregnant had lower levels of MiBP, a metabolite of DiBP, in comparison to participants in the rarely/never category. Migration of DiBP into food can occur from printing inks of food packaging via the “set off” effect, where the printed surface and its components contaminate the non-printed surface that ultimately comes into contact with food [36,37]. Further, a recent source study conducted by Bradley *et al.* (2013) provided evidence for the migration of DiBP into food through contact with recycled paperboard [16]. Therefore following a practice which eliminates packaging also appears to decrease the potential for DiBP contamination. In comparison to rarely/never eating frozen fruits and vegetables during pregnancy, participants that reported sometimes following this practice had higher MBzP levels. This result seems plausible since potential sources for BzBP are present in the frozen food process as conveyor belts and packaging; however the respondents who endorsed always/usually consuming frozen fruits and vegetables did not have higher MBzP concentrations [17,38]. Future research should investigate the role of microwaveable packaging for frozen vegetables as another source of phthalates.

It is important to consider that non-dietary behaviors may have impacted the relationship between food consumption practices and some metabolite levels since DEP, DiBP and DnBP have sources in personal care and household products as well as the diet. Indeed, a majority of respondents that always/usually used ecofriendly PCPs (73.5%) and ecofriendly house products (73.8%) also reported always/usually consuming ecofriendly food products. Future research should consider a factor analysis approach to create “behavior profiles” that may distinguish between low exposure versus high exposure practices.

According to a National Toxicology Program (NTP) assessment, 90% of DEHP exposure is estimated to occur through dietary sources [39]. Therefore, we expected to observe the greatest impact of food consumption practices on the metabolite levels of this particular phthalate species. Surprisingly, rarely or never eating food marked “organic”, “pesticide-free”, or “chemical-free” during pregnancy was associated with lower concentrations of the sum of DEHP metabolites in comparison to always/usually following this practice while other dietary relationships tested were null. According to the United States Department of Agriculture (USDA), an organic product is defined as one that is grown without the application of conventional pesticides, antibiotics or growth hormones and by farmers who emphasize the conservation and use of natural and renewable resources [40]. In this definition, the use of DEHP-containing materials such as PVC tubing and packaging is not necessarily excluded, leaving the opportunity for DEHP contamination in organic food. Another distinct explanation for these null results is that in recent years food industries have reduced the use of DEHP in products thus decreasing concentrations in foods. In fact, since 2008 the European Commission has limited phthalates, including DEHP, in food contact materials made of plastic [41].

There appears to be some support for the development of exposure reducing interventions for DEP, DiBP and BzBP that focus on behavioral changes, particularly the selection of natural and minimally processed products. However, the feasibility of adopting these practices in the general population should be carefully considered since environmental health-related behaviors have been found to vary in relation to sociodemographic factors [22].

Strengths of this study include the examination of dietary phthalate exposures in a population of pregnant women with a large sample size. This is also the first study to investigate the hypothesis that variation in consumer practices may impact phthalate exposure and that those promoting use of homegrown and ecofriendly products may reduce such exposures. Use of biomarkers allowed for the accurate measurement of internal dose thereby reducing phthalate exposure misclassification. Further, questionnaire data on significant covariates were included as confounder variables in models to provide more accurate regression estimates.

Limitations of our study include the reporting of food frequency and consumption behaviors on questionnaires days or even weeks after initial visits when urine specimens were collected. While questions asked about “typical” behaviors, metabolites reflect very recent exposures which may, for some respondents, not have been “typical”. In the future, we would be more intentional about wording and ask about behaviors in the 24 h prior to urine collection. Further, serving sizes were not defined for the food groups consumed per week resulting in a crude estimate of dietary consumption. Such misclassification is expected to be non-differential and would serve to bias the association toward the null. Behavior questions that were phrased in a vague manner may have led to exposure misclassification since each woman had individual interpretations of the consumption behaviors queried. In the future, we would consult with a behavioral methods specialist in creating these types of questions. We did not quantify the number or the amount of products that were consumed within each of the practices. Therefore it is possible that women whom reportedly made ecofriendly purchases may have had lower phthalate concentrations due to decreased use or amount of products rather than the selection for chemical-free alternatives. Further, the cohort was not representative of the U.S. population in terms of education since 43.6% of participants had some graduate school or graduate degree as compared to 9.9% in the United States as a whole and 13.3% had a high school degree or less compared to 42.4% [42,43]. In addition, the study only focused on English speakers. However, our study population had comparable concentrations to the current NHANES report. Use and thus exposure of diisononyl phthalate (DiNP) and diisodecyl phthalate (DiDP) increased significantly over the last decade in the United States causing concern given recent evidence of the toxicological potential of DiNP [33,44]. A limitation of our study is that the majority of our population lacked data on these phthalate species. However, a sub-analysis of 360 samples revealed a statistically significant positive association between the consumption of fast food and concentrations of the DiNP metabolite (β: 0.07; 95% CI: 0.001–0.13). Future research that seeks to understand the sources of these particular phthalate species will be of importance as they replace DEHP. Lastly, it is possible that the observed associations were due to chance since multiple testing increases the probability of false positives (type I errors) and analyses were not adjusted (to avoid increasing the probability of false negatives). These results should be confirmed in future studies.

## 5. Conclusions

This study examined the association between intake of specific foods and phthalate metabolite concentrations in a cohort of pregnant women. We observed negative associations between the consumption of spices and MiBP, MnBP, MBzP and the sum of DEHP metabolite concentrations. Intake of dairy was related to lower MiBP and sum of DEHP metabolites, and we also observed an increase in MEP exposure with dairy consumption. These unexpected results may be due to an inability to obtain exposure history within 24–48 h of urine sample collection. Therefore, future work on dietary phthalate exposure utilizing 24-h dietary recalls is needed to identify foods that contribute to biomarker levels in pregnant women. This is also the first study to evaluate the relationship between self-reported consumer practices and phthalate metabolite concentrations, thus informing future research on behavioral interventions to reduce pregnant women’s exposure to phthalates. We observed that reported use of ecofriendly, chemical-free and environmentally friendly personal care and household products was related to lower levels of MEP while consumption of homegrown foods resulted in lower MEP and MiBP concentrations. Limiting frozen fruits and vegetables was associated with lower MBzP levels. These results support the need for further discernment of the role of particular consumer practices in reducing phthalate exposures particularly during pregnancy. Future research on this topic should clearly define the types of products purchased, the amount and frequency of use as well as the impact of adhering to multiple consumer behaviors simultaneously. The feasibility of adopting these practices in the general population should also be carefully considered.

## Supplementary Files

Supplementary File 1 Supplementary Information (PDF, 128 KB)
