# Supplementary material for: Dietary Phthalate Exposure in Pregnant Women and the Impact of Consumer Practices
Source: Int J Environ Res Public Health. 2014 Jun 12;11(6):6193–215. doi: 10.3390/ijerph110606193 (PMC4078574; doi:10.3390/ijerph110606193)
Supplement: Supplementary File 1 — Supplementary Information (PDF, 128 KB) [file ijerph-11-06193-s001.pdf]

Supplementary Information

Dietary Phthalate Exposure in Pregnant Women and the Impact of Consumer Practices

---

Food Consumption and Consumer Practice Questions in the Tides First Trimester Questionnaire

Questions 13 through 21 are about your lifestyle and diet.

The following questions are about the kinds of products and foods you choose to buy and consume and why you choose them. Select one answer per question.

15. When I buy personal care products (such as cosmetics and toiletries), I try to make sure they are ecofriendly, chemical-free or environmentally friendly.

- ☐ Always
- ☐ Usually
- ☐ Sometimes
- ☐ Rarely
- ☐ Never

16. When I buy things for my home (such as household products, furniture, and upholstery), I try to make sure they are ecofriendly, chemical-free or environmentally friendly.

- ☐ Always
- ☐ Usually
- ☐ Sometimes
- ☐ Rarely
- ☐ Never

17. When I consume food, I try to make sure it is organic, ecofriendly, chemical-free or environmentally friendly.

- ☐ Always
- ☐ Usually
- ☐ Sometimes
- ☐ Rarely
- ☐ Never

20. How often do you try to make sure that the food and beverages you consume come in plastics that you think are safe?

- ☐ Always
- ☐ Usually
- ☐ Sometimes
- ☐ Rarely
- ☐ Never
- ☐ Don't Know

21. How often do you check the recycling code on the bottle?

- ☐ Always
- ☐ Usually
- ☐ Sometimes
- ☐ Rarely
- ☐ Never

Questions 22 through 25c, are about your typical diet. For each one, choose <u>one</u> answer from among the following options: always, usually, sometimes, rarely, never, don't know.

22. Since you became pregnant, how often have you consumed foods marked "organic", "pesticide-free", or "chemical-free"?

- ☐ Always
- ☐ Usually
- ☐ Sometimes
- ☐ Rarely
- ☐ Never
- ☐ Don't Know

23. Since you became pregnant, how often have you consumed food that is grown, raised, or caught by you or your family and friends (as opposed to bought in a store)?

- ☐ Always
- ☐ Usually
- ☐ Sometimes
- ☐ Rarely
- ☐ Never

- ☐ Don't Know

24. Since you became pregnant, how often have you consumed food that is unprocessed (as opposed to canned, frozen, dried, cooked, processed, pre-prepared, or packaged)?

- ☐ Always
- ☐ Usually
- ☐ Sometimes
- ☐ Rarely
- ☐ Never
- ☐ Don't Know

25a. Since you became pregnant, when you consume fruit or vegetables, how often are they <u>canned</u>?

- ☐ Always
- ☐ Usually
- ☐ Sometimes
- ☐ Rarely
- ☐ Never
- ☐ Don't Know

25b. Since you became pregnant, when you consume fruit or vegetables, how often are they <u>frozen</u>?

- ☐ Always
- ☐ Usually
- ☐ Sometimes
- ☐ Rarely
- ☐ Never
- ☐ Don't Know

25c. Since you became pregnant, when you consume fruit or vegetables, how often are they fresh?

- ☐ Always
- ☐ Usually
- ☐ Sometimes
- ☐ Rarely
- ☐ Never
- ☐ Don't Know

26. Now think about the foods you've eaten in a typical week. For each item, please estimate the number of servings eaten in a typical week.

(a)	peanut butter (or other nut butters)?	_______ servings in a typical week
(b)	beef?	_______ servings in a typical week
(c)	seafood?	_______ servings in a typical week
(d)	poultry (such as chicken, turkey, *etc.*…)?	_______ servings in a typical week
(e)	other meats (such as pork, organ meats, lamb, *etc.*…)?	_______ servings in a typical week
(f)	spices (including salt/pepper, dried herbs, and other seasonings)?	_______ servings in a typical week
(g)	oils, butter, lard, or shortening?	_______ servings in a typical week
(h)	Soy products (soy milk, tofu, soy nuts, edamame, soy protein, *etc.*…)	_______ servings in a typical week
(i)	Dairy products (milk, cheese, yogurt, cottage cheese, ice cream, *etc.*…)	_______ servings in a typical week

27. In a typical **week** since you became pregnant how many meals have you eaten in a restaurant, had take-out food, or had food delivered?

_______ meals

If your answer to 27 was greater than 0, please answer 27a:

27a. How many of these meals are typically fast food?

_______ meals

28. In a typical **week** since you became pregnant, how many times per **day** have you had at least a serving of a beverage that comes in a ***plastic bottle***? (include water, soda, juice, milk, and any other drink you can think of. Also, include drinks that were poured out of large plastic bottles as well as drinks from individual bottles.)

_______ drinks/day

29. In a typical **week** since you became pregnant, how many times per **day** have you had at least a serving of a beverage that comes in a ***can***? (include soda, juice, and any other drink you can think of. Also, include drinks that were poured out of large cans as well as drinks from individual cans).

_______ drinks/day

© 2014 by the authors; licensee MDPI, Basel, Switzerland. This article is an open access article distributed under the terms and conditions of the Creative Commons Attribution license (http://creativecommons.org/licenses/by/3.0/).
